# Extract of *Indigofera spicata* Exerts Antiproliferative Effects on Human Colorectal and Ovarian Carcinoma Cells

**DOI:** 10.3390/toxins17090431

**Published:** 2025-08-29

**Authors:** Galyna Shuvayeva, Mykola Tupychak, Olena Vovk, Dmytro Demash, Svitlana Chernyshuk, Yaroslav Bobak, Andriy Prokopiv, Nazariy Pokhodylo, Leoni A. Kunz-Schughart, Mary T. Fletcher, Oleh Stasyk

**Affiliations:** 1 Institute of Cell Biology, National Academy of Sciences of Ukraine, Drahomanov Str., 14/16, 79005 Lviv, Ukraine; shuvayeva77@gmail.com (G.S.); vovkoliv@gmail.com (O.V.); ddemash@gmail.com (D.D.); s.chernyshu@ukr.net (S.C.); bobakyaroslav@gmail.com (Y.B.); 2 Department of Organic Chemistry, Chemical Faculty, Ivan Franko National University of Lviv, Kyryla and Mefodiya Str., 6, 79005 Lviv, Ukraine; tupychakmykola@gmail.com (M.T.); pokhodylo@gmail.com (N.P.); 3 Department of Botany, Biological Faculty, Ivan Franko National University of Lviv, M. Hrushevskoho Str., 4, 79005 Lviv, Ukraine; prokopivandriy1@gmail.com; 4 Botanic Garden, Ivan Franko National University in Lviv, Cheremshyny Str., 4, 79014 Lviv, Ukraine; 5 Department of Pharmacy and Biology, Stepan Gzhytskyi National University of Veterinary Medicine and Biotechnologies Lviv, Pekarska Str., 50, 79010 Lviv, Ukraine; 6 OncoRay—National Center for Radiation Research in Oncology, Faculty of Medicine, University Hospital Carl Gustav Carus, TUD Dresden University of Technology and Helmholtz-Zentrum Dresden-Rossendorf, Fetscherstr 74, 01307 Dresden, Germany; leoni.kunz-schughart@oncoray.de; 7 National Center for Tumor Diseases, Partner Site Dresden (NCT), 01307 Dresden, Germany; 8 Queensland Alliance for Agriculture and Food Innovation (QAAFI), The University of Queensland, P.O. Box 156, Archerfield 4108, Australia; mary.fletcher@uq.edu.au; 9 Institute of Epidemiology and Hygiene, Danylo Halytsky Lviv National Medical University, Zelena Str., 12, 79005 Lviv, Ukraine

**Keywords:** indospicine, *Indigofera spicata*, metabolic anticancer therapy, arginine deprivation

## Abstract

Metabolic anticancer therapy based on enzymatic arginine (Arg) deprivation (ADT) is currently being evaluated in clinical trials. The combination of ADT with low doses of the plant cytotoxic analogs of Arg, canavanine (Cav) or indospicine (Isp), have been proposed as being more efficient and selective against malignant cells. The leguminous plant *Indigofera spicata* contains one of the highest known amounts of Isp. Here we demonstrate for the first time that the Isp-containing ethanolic extract from *I. spicata* is growth-inhibiting and toxic for cultured human colorectal and ovarian carcinoma cells. The extract reduces the viability of colorectal carcinoma cells two-fold under Arg-deficient conditions and entirely abrogates their residual proliferative potential (growth recovery) after the treatment. Pre-exposure of the extract to recombinant human arginase I (rhARGI) as a therapeutic Arg-depleting agent did not impact the extract’s efficacy. Further development of Isp as a component of combinatorial anticancer metabolic targeting strategies is discussed.

## 1. Introduction

Arginine (Arg) is a functionally versatile amino acid and is semi-essential in humans [1]. Arg is a potent activator of mTOR signaling and is required to synthesize important secondary signaling molecules, such as nitric oxide, polyamines, proline, creatinine, and agmatine [2,3]. Somewhat paradoxically, genetic alterations during malignant transformation often lead to the deficiency of cancer cells in Arg biosynthesis and a profoundly elevated uptake of exogenous Arg [2]. Such a dependency makes some malignant cells sensitive to Arg deprivation therapy (ADT), which is based on recombinant Arg-degrading enzymes, human arginase I (rhARGI) or bacterial arginine deiminase (ADI), which deplete Arg in the bloodstream [4,5,6]. Notably, the results of animal studies and clinical trials suggested that ADT as a monotherapy inhibits the growth of auxotrophic for Arg, i.e., is deficient in argininosuccinate synthetase (ASS1) tumors, but by itself is insufficient for eliminating cancer cells in vivo [7,8,9,10,11,12,13]. After terminating the administration of Arg-degrading enzymes, the physiological Arg level in the bloodstream is rapidly restored, and the proliferation of surviving or dormant malignant cells resumes [5]. To improve ADT efficacy, we and others proposed combinational approaches that comprise Arg-degrading enzymes and rationally chosen chemotherapeutic drugs [3,11,12,13].

We have previously reported on the selective anticancer effect of low micromolar doses of canavanine (Cav), a structural Arg analog of plant origin (e.g., from *Canavalia ensiformis*), when rhARGI concomitantly deprives Arg [14]. The Cav cytotoxic effect depends on its elevated uptake by cancer cells, specific dysregulation of the mTOR/eiF2α signaling, Cav misincorporation into nascent proteins instead of Arg, exacerbation of ER stress, and progression of apoptosis [14,15]. The rhARGI/Cav combination was effective against various cancer cells grown in 2D or 3D culture, including those relatively resistant to ADT mono-treatment [15]. Arg-starved pseudonormal cells (either in formulated Arg-free medium (AFM) or exposed to rhARGI-supplemented Arg-rich complete medium (CM) were much more resistant to this treatment than the malignant cell populations. However, elucidation of Cav pharmacokinetics in vivo requires additional, more detailed studies, as it remains a non-preferred substrate of rhARGI [16].

Therefore, we searched for an alternative Arg analog in plants that is unlikely to be degraded by rhARGI and identified indospicine. L-indospicine (2(S)-2,7-diamino-7-iminoheptenic acid, Isp) is a natural Arg proteomimetic analog which differs from Arg by a single substitution of the nitrogen atom to carbon in position 6 of its backbone (Figure S1) [17].

Isp is found only in leguminous subtropical plants of the genus *Indigofera* and has previously been implicated as a cause of horse and camel meat toxicity for domestic dogs in Australia [18]. We have also reported that chemically synthesized Isp exerts selective antiproliferative and cytotoxic effects on cultured colorectal carcinoma cells in combination with rhARGI. Notably, in in vitro experiments, the effective cytotoxic Isp concentrations were in the low micromolar range for colorectal cancer cells and about 40 times higher for cultured pseudonormal human cells [16]. In Arg-deprived cancer cells, Isp deregulated the PI3K-Akt-mTOR pathway, exacerbated endoplasmic reticulum (ER) stress, and triggered profound caspase-dependent apoptosis. These effects were dependent on protein synthesis de novo, suggesting Isp is actively incorporated into nascent proteins. During co-exposure, rhARGI neither degraded Isp nor was its activity inhibited by Isp [16]. This preliminary data warrants further studies on Isp as an anticancer agent in the frame of ADT.

Unfortunately, pure Isp is not readily available, neither through synthesis nor isolation from plant sources. However, we hypothesized that Isp-containing plant extracts may serve as a readily available substitute for pure Isp, which may for instance be applied for treating localized or physically accessible tumors. Isp has been found in a number of leguminous *Indigofera* plants of subtropical and tropical origin, with the highest levels detected in *I. spicata* (creeping indigo) and *I. linnaei* (Birdsville indigo) [18,19]. Whilst *I. spicata* has been cultivated and introduced throughout various subtropical and tropical regions before its toxicity became apparent [19], *I. linnaei* has a somewhat limited spread, occurring naturally in inland arid areas of Australia.

Here, we report for the first time that crude Isp-bearing ethanolic extracts of the perennial legume *I. spicata* exert a strong antiproliferative effect in cultured human colorectal and ovarian carcinoma cells when simultaneously applied with rhARGI.

## 2. Results

### 2.1. Preparation of the Isp-Containing Extracts from Dried Biomass (Seedpods) of I. spicata

**Figure 1:**
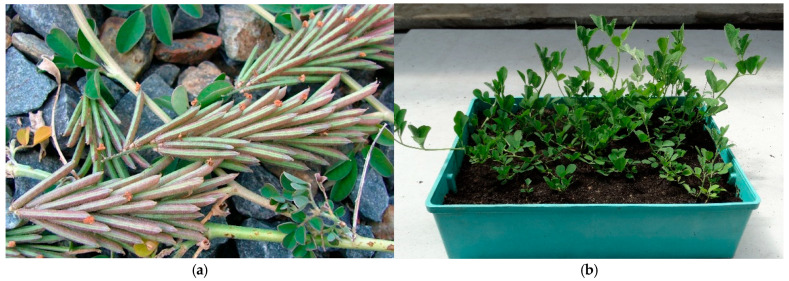
*I. spicata* seedpods [24] (**a**); *I. spicata* grown from seeds under greenhouse conditions (**b**).

Mature *I. spicata* plants collected in the Brisbane area (Queensland, Australia) were used as an Isp source in this study. Species identification was conducted according to Plants of the World Online (POWO) [20], Australian Plant Census (APC) Council of Heads of Australian Herbaria [21] and *Indigofera* revision in Australia [22]. Such *Indigofera* plant material has previously been shown to consistently produce and accumulate significant amounts of free Isp (0.4–1.5 mg Isp per g of dry biomass) [19,23]. The plant material used in this study was collected by Prof. Mary Fletcher, with both immature green and mature brown intact seedpods separated and air-dried, and then transferred to the Botanical Garden of Lviv National University (Ukraine). Mature brown seedpods were reserved to grow *I. spicata* under greenhouse conditions as an alternate source of biomass for future studies (Figure 1).

**Figure 2:**
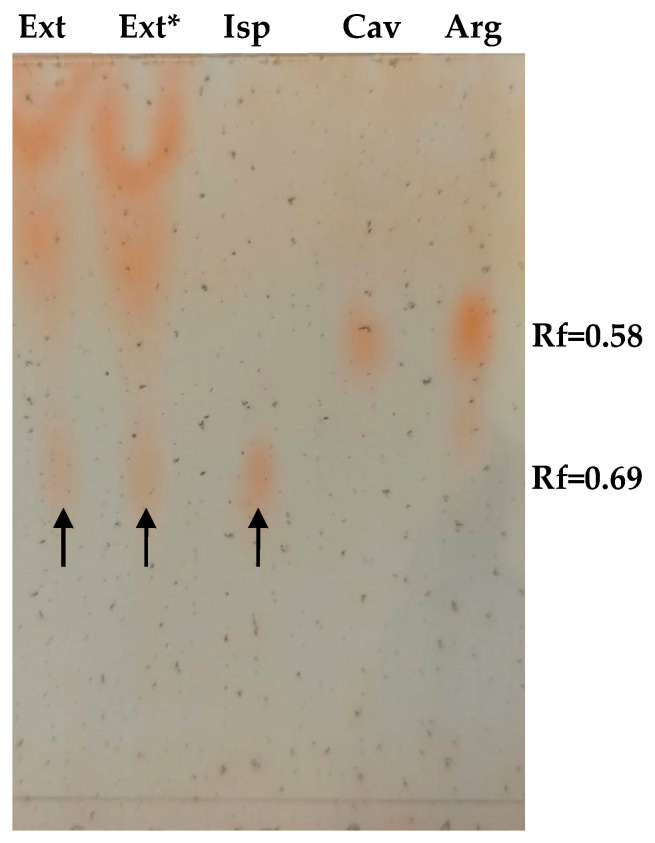
Thin layer chromatographic visualization of Isp in an *I. spicata* extract. 10 µL of extract and 1.5 µg of amino acids standards (Isp, Cav, and Arg) were loaded as indicated. Ext*—designates *I. spicata* extract pre-treated with 2 U/mL of rhARGI for 30 min. Spots corresponding to Isp are designated with arrows.

*I. spicata* dried plant material was utilized to provide an ethanolic extract for employment in all cell-based experiments described herein (for details, see Materials and Methods). Thin-layer chromatography followed by treatment with ninhydrin confirmed the presence of Isp in this extract according to [25,26] (Figure 2).

**Figure 3:**
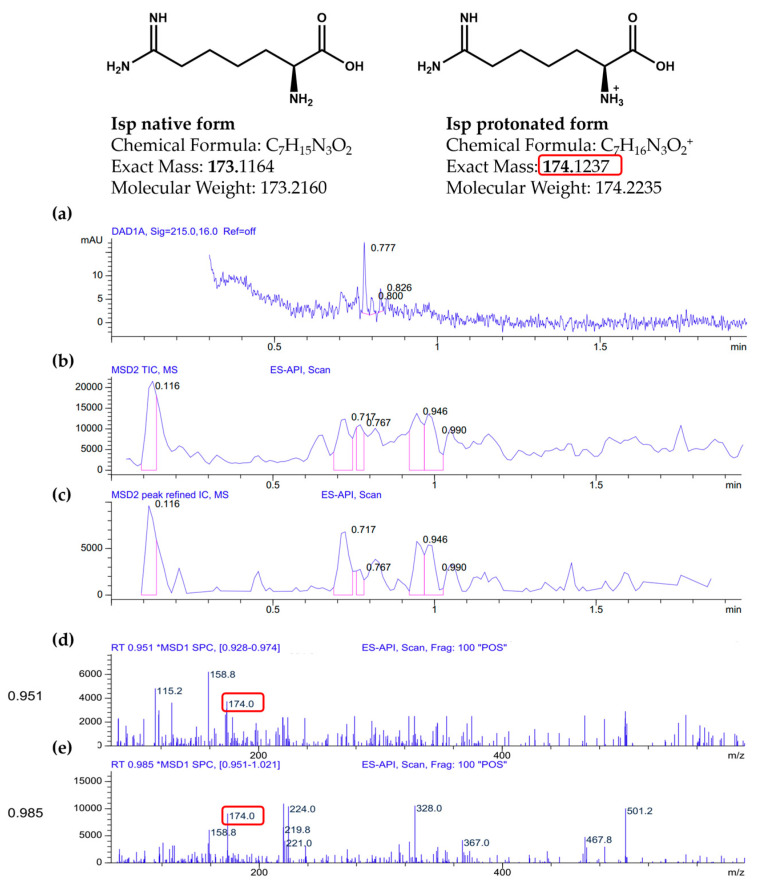
LC-MS analysis of *Indigofera spicata* extract (the MS peaks corresponding to Isp are designated with red square): (**a**) a UV chromatogram from the Diode Array Detector (DAD) at 215.0 nm with a 16.0 nm bandwidth and no reference wavelength (“Ref = off”); (**b**) a Total Ion Chromatogram (TIC) from the Mass Selective Detector (MSD) using Electrospray Atmospheric Pressure Ionization (ES-API); (**c**) a refined Extracted Ion Chromatogram (IC) for *m*/*z* 174.0 with software-enhanced peak processing; (**d**) a mass spectrum extracted from the retention time interval 0.928–0.974 min, showing a prominent ion at *m*/*z* 174.0; (**e**) a second mass spectrum from 0.951–1.021 min, also showing *m*/*z* 174.0.

We observed a purple/violet TLC spot at Rf = 0.69 after ninhydrin visualisation, corresponding to co-eluting standard Isp (Figure 3). As predicted, the treatment of the extract with 2 U/mL of rhARGI did not result in Isp degradation, with the intensity of the Isp TLC spot in the Ext* extract being unchanged. Notably, minor purple/violet spots at Rf = 0.58, tentatively corresponding to Cav and Arg, became visible in the lanes with the loaded extract (Figure 2). Comparison of the relative intensity of the Isp TLC spot derived from 10 µL of the *I. spicata* seedpod extract with that of 1.5 µg of synthetic Isp standard (Figure 2) enabled the Isp concentration of the extract to be estimated. In this regard, ninhydrin derivatisation appears useful for both identification (comparison of Rf) and quantitation/semi-quantitation of free amino acids in various products [27,28]. Thereby, we predicted the Isp concentration in the extract to be in the range of 1 mM. Such a concentration is in accord with previously reported Isp content in the biomass of *I.spicata* collected in similar regions of Australia [19,23,29]. The presence of Isp in the *I. spicata* seedpod extract was further confirmed by our LC-MS analysis, identified as a molecular ion peak [M+1] at 174 Da (Figure 3).

### 2.2. Impact of I. spicata Extract on the Viability of Human Carcinoma Cells

**Figure 4:**
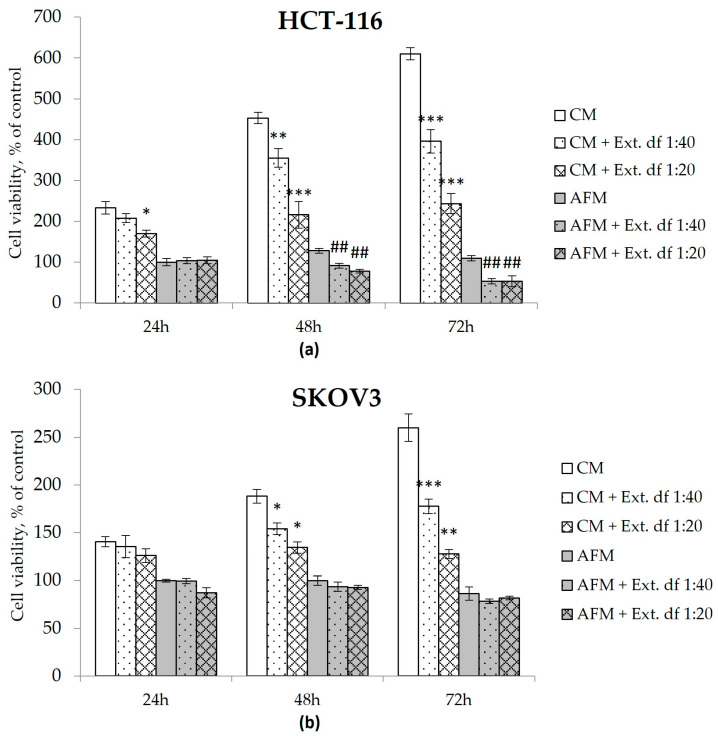
Cell viability of human colorectal carcinoma HCT-116 (**a**) and ovarian carcinoma SKOV3 (**b**) cells determined via the MTT assay. Cells were exposed to Arg-rich complete (CM) or Arg-free (AFM) media with or without an Isp-containing extract of *I. spicata* (Ext.) prepared as described in Materials & Methods. The treatment time corresponds to the analytical timepoints at 24, 48 and 72 h as indicated. Two dilutions of the extract were applied that correspond to final concentrations of Isp of ~25 µM (df (dilution factor) 1:40) and 50 µM (df 1:20), respectively. Data show relative values to the measurements before treatment (control cells in timepoint 0) set to 100% and are presented as mean ± SD of three independent experiments. (* *p* ≥ 0.05, ** *p* ≥ 0.01, *** *p* ≥ 0.001 versus the CM group at the corresponding timepoint; # *p* ≥ 0.05, ## *p* ≥ 0.01, ### *p* ≥ 0.001 versus the AFM group at the corresponding timepoint).

Human colorectal carcinoma HCT-116 and ovarian carcinoma SKOV3 cells were applied as experimental models for assessing the cytotoxicity of the *I. spicata* extract (Figure 4). Both HCT-116 and SKOV3 cells were documented earlier to be relatively resistant to ADT mono-treatment, i.e., they did not exhibit significant loss of viability and concomitant apoptosis when starved for Arg [15,16,30]. Simultaneously, as shown in our previous works, all cultured cancerous cells cease proliferation in Arg-deficient medium in the absence of citrulline as they cannot synthesize Arg de novo due to the lack of expression of the urea cycle enzyme ornithine transcarbamylase (OTC), which converts ornithine to citrulline [14,30]. We chose extract dilutions of 1:40 and 1:20 in PBS corresponding to roughly 25 and 50 µM Isp, respectively, to mimic concentrations close to the cytotoxic IC_50_ values for chemically synthesized Isp in Arg-starved HCT-116 cells [16].

*I. spicata* extract evoked an additional adverse effect on the viability of HCT-116 cells in AFM, e.g., a 50% reduction in the relative viable cell count was observed after 72 h of the combination treatment at both tested extract dilutions (Figure 4). SKOV3 cells did not exhibit a similar response in AFM. However, contrary to pure Cav or Isp [16], the *I. spicata* extract inhibited culture growth of both tested cell lines even when administered in the presence of Arg, exerting a time- and dose-dependent cytotoxic effect in CM medium. Here, the 1:40 extract dilution exposed for 72 h inhibited growth in both cell lines, resulting in a relative viable cell count reduced to 35% of untreated controls, while the 1:20 extract dilution completely stopped proliferation of HCT-116 and SCOV3 cells (Figure 4). Notably, no additive adverse effect was observed when the *I. spicata* extract was supplemented to cells starved for Lysine, an alternative essential amino acid (Figure S2).

### 2.3. Effect of I. spicata Extract on the Residual Proliferative Potential of Cancer Cells

**Figure 5:**
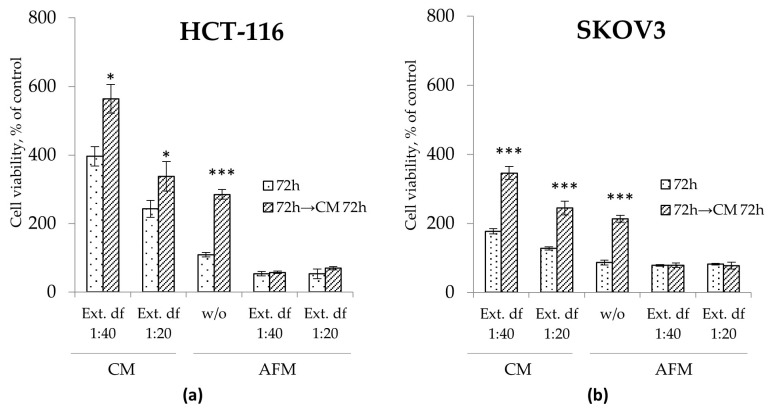
Residual proliferative potential assessed via MTT assays in HCT-116 (**a**) and SKOV3 (**b**) cells treated with or without (*w*/*o*) *I. spicata* extract (Ext.) in Arg-rich complete (CM) and Arg-free (AFM) media for 72 h. After termination of the treatment, cells were allowed to grow in freshly replaced CM for an additional 72 h. Two dilutions of the extract were applied corresponding to final concentrations of Isp of 25 µM (df (dilution factor) 1:40) and 50 µM (df 1:20), respectively. Data show relative values to the measurements before treatment (control cells at timepoint 0 set to 100%) and are presented as mean ± SD of three independent experiments (* *p* ≥ 0.05, ** *p* ≥ 0.01, *** *p* ≥ 0.001 versus control cells for each group).

One of the aspects of the cancer cells’ sensitivity to ADT in vitro is their ability to restore proliferation when AFM medium is replaced by fresh Arg-containing CM, reflecting their “residual proliferative potential”. To evaluate this capacity for growth recovery, cells were exposed to the various treatment conditions for 72 h, followed by the substitution of the supernatant with Arg-rich CM for an additional 72 h, post-treatment exposure time. As seen in Figure 5, treatment of the cancer cells with the *I. spicata* extract in CM, i.e., in the presence of Arg, led to some decrease in post-treatment proliferative potential. The analogous treatment with ADT, i.e., *I. spicata* extract in AFM, completely abrogated any residual proliferative potential in both cell models; in contrast, cells subjected to mono-ADT showed a 2.5-fold increase in cell numbers 72 h after CM substitution (Figure 5). The extract thus exerted a strong and persistent antiproliferative effect in AFM even at the highest tested dilution (1:40), corresponding to 25 µM Isp.

### 2.4. Antiproliferative Effect of I. spicata Extract in Combination with rhARGI

**Figure 6:**
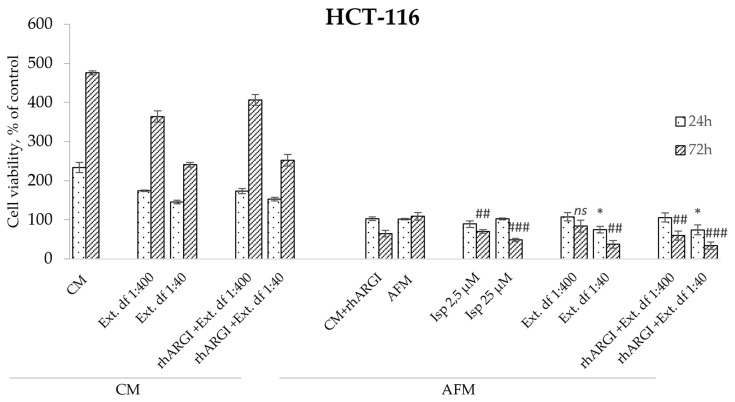
Relative viability of HCT-116 colorectal carcinoma cells exposed to rhARGI-pretreated and non-pretreated extracts of *I. spicata* in CM and AFM conditions. Cell viability was determined via the MTT assay. The exposure (treatment) periods and analytical timepoints were 24 and 72 h. Two dilutions of the extracts were applied corresponding to final Isp concentrations of ~2.5 µM (df (dilution factor) 1:400) and 25 µM (df 1:40), respectively. Pure indospicine (Isp) was used as a positive control to compare with the *I. spicata* extracts. Data show relative values to the measurements before treatment (control cells at timepoint 0 set to 100%) and are presented as means ± SD of three independent experiments (* *p* ≥ 0.05, ** *p* ≥ 0.01,*** *p* ≥ 0.001 versus AFM 24 h; # *p* ≥ 0.05, ## *p* ≥ 0.01, ### *p* ≥ 0.001 versus AFM 72 h; *ns* indicates not significant).

Finally, we addressed whether the *I. spicata* extract preserved its antiproliferative effects when administered in combination with rhARGI as an ADT agent. As shown in Figure 6, our rhARGI preparation was efficient in degrading Arg in CM, as growth of НСТ-116 cells was arrested under such conditions. The application of an rhARGI-pretreated *I. spicata* extract to cells retained clear and strong antiproliferative efficacy in both CM and AFM conditions. This becomes evident when comparing the cell viability data upon expose to rhARGI-pretreated 1:40 and 1:400 extract dilutions with the respective non-pretreated extracts (Figure 6). At a dilution of 1:40, treated and untreated extracts evoked similar inhibition of cell proliferation of approximately 70% at the 72 h timepoint. Interestingly, the plant extract induced a more profound drop in cell viability relative to pure Isp at a 25 µM concentration: the difference of 30% was already seen at 24 h of the treatment. This observation and the extract’s effects in an Arg-rich CM milieu suggest that components other than Isp may contribute to the extract’s cytotoxicity.

## 3. Discussion

We have reported in previous studies that Isp is selectively toxic in mid-micromolar doses for malignant cells when cultured as monolayers in medium depleted of Arg [16]. Our data also demonstrated that the specific anticancer effects of Isp and the more intensively studied Cav are mainly due to their incorporation into nascent proteins in place of Arg, thereby destabilizing their structure and function [14,16]. However, contrary to Cav, Isp is neither an rhARGI catabolic substrate nor its inhibitor at higher concentrations [16]. Therefore, Isp is hypothesized to be a more suitable component in combination treatment modalities with this recombinant enzyme, which is currently being evaluated in the clinics as an ADT agent in monotherapy or in combination with chemotherapeutic drugs [10,31,32,33,34,35]. However, chemically synthesized Isp is an expensive and not readily available compound.

In the present work, we aimed to determine whether amino acid-bearing ethanolic extracts of *I. spicata*, a leguminous plant reported to produce and accumulate free Isp in its biomass, will exert an antiproliferative effect in the presence of rhARGI, thus providing an affordable substitute for chemically synthesized Isp, at least for localized, accessible tumors. We have demonstrated here for the first time that the amino acid-containing extract of *I. spicata* exerts a strong cytotoxic and antiproliferative efficacy against cultured human colorectal and ovarian carcinoma cells. The antiproliferative effect was evident in both Arg-free (growth arrest and decreased ability to resume growth) and Arg-rich, complete medium (decreased growth rate), suggesting that the extract contains other antineoplastic compounds besides the proteomimetic Arg analogs, Isp and Cav [19].

Consistent with previous literature reporting that seeds of *I. spicata* contained appreciable concentrations of Isp [36], our thin layer chromatography and LC-MS analyses confirmed the presence of Isp in intact seedpod extracts. A more detailed analysis of the extract composition will follow shortly to identify and determine the nature of other putative compounds involved in the observed anticancer effects. It would be of interest to apply metabolomics studies with *I. spicata* extracts to get a deeper insight into the extracts’ constituents and their cellular effects [37,38].

Notably, pre-treatment of the extract with rhARGI did not alter the extract’s antiproliferative efficacy in our model cancer cells, supporting the notion that the approach offers promise for therapeutic combination. Indeed, our data suggest that the *I. spicata* extract, similarly to pure Isp, may be efficient in enhancing rhARGI-based ADT. Moreover, our recent preliminary data extends the list of tumors responsive to such a combination modality to head and neck squamous cell carcinomas and prostate carcinomas, suggesting the observed effects may be of a general, cell-type-independent character.

Importantly, exposure to *I. spicata* extracts in an Arg-deficient medium resulted in the inability of human colorectal and ovarian carcinoma cells to recover growth upon treatment completion, i.e., failure to resume normal proliferative capacity when Arg is re-supplemented with fresh CM. This suggests that ADT, which partially overlaps in time with treatment with Arg cytotoxic analogs, may prevent malignant cells from regrowing, while possibly reducing overall toxicity. We recognize the limitation of this study as only in vitro models have been evaluated. Our follow-up studies will address these issues and also assess whether the treatment with Isp or *I. spicata* extract prolongs the disease-related survival of laboratory animals with transplanted model carcinomas and alternative tumors. Another experimental task will be to purify sufficient quantities of Isp from *I. spicata* material for such studies. In this context, we aim to prove the feasibility of ion-exchange chromatography-based methods.

As mentioned above, since pure Isp is difficult to obtain, our data emphasize an interesting and reasonable alternative with cancericidal efficacy in the form of *I. spicata* extracts (or, potentially, extracts from *I. linnaei*, which is not as common outside of Australia but has a similar high Isp content [19]). *I. spicata* exhibits a widespread distribution in tropical countries as both native and introduced species (see Plants of the World Online [20]. Our experience suggests it is also relatively easy to cultivate in greenhouse conditions (Figure 1) and represents a viable cultivation industry of herbal medicine.

## 4. Conclusions

In this article, we evaluated the antiproliferative effects of an ethanolic extract of the leguminous plant *I. spicata* containing Isp on cultured human colorectal and ovarian carcinoma model cells. We suggest that such an extract can be a convenient substitute for pure Isp as a component of metabolic anticancer therapy based on Arg deprivation after follow-up studies on additional cell and animal models.

## 5. Materials and Methods

### 5.1. Reagents

Dulbecco’s Modified Eagle Medium (DMEM), selective amino acids-deficient DMEM, fetal bovine serum (FBS), dialyzed FBS, gentamycin, L-arginine monohydrochloride (Arg), L-methionine, L-leucine, and L-lysine monohydrochloride were purchased from Sigma-Aldrich (St. Louis, MO, USA), PAN-Biotech (Aidenbach, Germany), CytivaHyClone (Logan, UT, USA), or Merck (Darmstadt, Germany). Pure indospicine was provided by Prof Fletcher, with the synthesis previously described [17]. All chemicals used in extraction and purification experiments were commercially available and of highest purity. The recombinant human arginase I (rhARGI) used in this study was expressed as a secretory protein in the methylotrophic yeast *Ogataea polymorpha* and affinity-purified in O. Stasyk’s laboratory.

### 5.2. Cell Lines and Culture Conditions

Human colorectal carcinoma HCT-116 and ovarian carcinoma SKOV3 cells used in this work were obtained from the ATCC (Manassas, VA, USA). All cell cultures tested negative for mycoplasma contamination with MycoAlert (Cambrex Bio Science Rockland, Inc, ME, USA) and PCR Mycoplasma Test (Applichem, Darmstadt, Germany) kits prior to use.

Cells were routinely incubated in DMEM supplemented with 10% FBS, 50 mg/l gentamicin and 2 мM glutamine at 37 °C in a humidified atmosphere with 5% CO_2_. Arg deprivation in the culture medium was achieved by specially formulated DMEM (CytivaHyClone, USA), selectively lacking Arg. Arg-free medium (AFM) and the respective Arg-rich complete medium (CM) were supplemented with 10% dialyzed FBS devoid of small molecules such as amino acids (<10 kDa cut off). All other required components, including glutamine and gentamycin, were added to AFM as to complete medium. Selective Arg deprivation by the application of AFM and CM + rhARGI preparation was shown previously to be identically effective in triggering growth arrest in various cultivated tumor cells [16]. Residual proliferative potential after starvation for Arg was estimated after replacing the Arg-deficient media with for fresh CM, and incubation of the cells for an additional 72 h to assess relative changes in cell numbers over time.

### 5.3. Cell Viability Detection by the MTT Assay

For the evaluation of the effect of various experimental (treatment) conditions on cell viability, cells were initially seeded at 2 × 10^4^ cells per well into 96-well culture plates. The cell viability assay was performed with MTT (3-(4,5-dimethylthiazol-2-yl)-2,5-diphenyltetrazolium bromide) reagent as described in a previous report, and cell viability and relative cell count, respectively, was presented in percent related to the levels of control cells at 0 h (before the onset of treatment).

### 5.4. Preparation of Plant Extracts from I. spicata

*I. spicata* plant material was collected at a single location in Brisbane area (Queensland, Australia) by Prof. M. Fletcher. A separate pressed voucher specimen was submitted to the Queensland Herbarium with the species identification confirmed as *I. spicata* (AQ 952785). The collected seeds from brown mature seed pods were used to establish plants growing under standard greenhouse conditions at the Botanical Garden of the Ivan Franko National University of Lviv. *I. spicata* intact green seedpods (containing seeds) was subjected to extraction to isolate an amino acids-containing fraction. For this purpose, 30 g of air-dried plant material (intact seedpods) was mechanically grounded to powder, diluted in a1.5 L solution of 70% EtOH /0.01M HCl and incubated at room temperature for 24 h. The extraction step was repeated thrice under the same conditions. The extraction was performed according to the previously described procedures with minor modifications [23].

The resulting extracts were filtered through Schott filters of different pore sizes and concentrated in rotary evaporator under lowered pressure and temperatures below 40 °C until the concentrated extract volume was around 2 mL. In the case of a low dispersed precipitate being observed, it was removed by centrifugation. The concentrated extract was diluted in PBS in a volumetric flask to a final constant volume (100 mL), filter sterilized and stored at 4 °C.

### 5.5. Thin Layer Chromatography of Extracts from I. spicata

Amino acids of interest were identified by thin layer chromatography (TLC) according to [25,26] with modifications, using aluminium plates covered with 0.2 mm silica gel 60 F_254_. Individual amino acid standards (Isp, Cav and Arg) or plant extracts were loaded on the start line of the chromatographic plate, and the plate was later dried at room temperature in a vacuum chamber (desiccator). The eluate was prepared by diluting 1 mL of glacial acetic acid in 90 mL of distilled water and tittering it to рН3.74 using 2 mM sodium acetate solution. The resulting buffer was mixed with acetonitrile (4% *v*/*v*). After the standard chromatographic procedure, the plate was dried and treated with 1% ninhydrin solution for amino acid visualization. The colored spots were compared with loaded controls of individual amino acids (Isp, Cav, and Arg).

### 5.6. LC-MS Analysis of Extracts from I. spicata

The analysis was performed using an Agilent 1100 LC/MSD system with a Poroshell 120 SB-C18 column (4.6 × 30 mm, 2.7 µm) at 60 °C. Mobile phase: water with 0.1% formic acid and acetonitrile with 0.1% formic acid, gradient from 1% to 100% B over 1.5 min. Scan range: 83–1000 *m*/*z*. The [M+H]^+^ ion (a) is observed at *m*/*z* 174.0, corresponding to the protonated form of Isp (C_7_H_16_N_3_O_2_^+^). The peak of Isp appears between 0.9 and 1.0 min, overlapping with other components. LC-MS spectra data are provided in Figure 3.

### 5.7. Statistical Analysis

Mean values ± SD from repeated, independent experiments (*n* ≥ 3) are shown in the Figures. Statistical analysis was performed using Student’s *t*-test. Differences in the means were considered significant when the calculated *p*-value was <0.05.

## Data Availability

The data presented in this study are available on request from the corresponding author due to legal restrictions that concern some of the dataset or materials.

## Supplementary Materials

The following supporting information can be downloaded at: https://www.mdpi.com/article/10.3390/toxins17090431/s1, Figure S1 Chemical structures of L-indospicine, Isp (left) and L-arginine, Arg (right). Figure S2 Cell viability of human colorectal carcinoma HCT-116 (a) and ovarian carcinoma SKOV3 (b) cells determined via the MTT assay.
