# Supplementary material for: Extract of Indigofera spicata Exerts Antiproliferative Effects on Human Colorectal and Ovarian Carcinoma Cells
Source: Toxins (Basel). 2025 Aug 29;17(9):431. doi: 10.3390/toxins17090431 (PMC12474036; doi:10.3390/toxins17090431)
Supplement: Supplementary file 1 [file toxins-17-00431-s001.zip › Figure S2_.pdf]

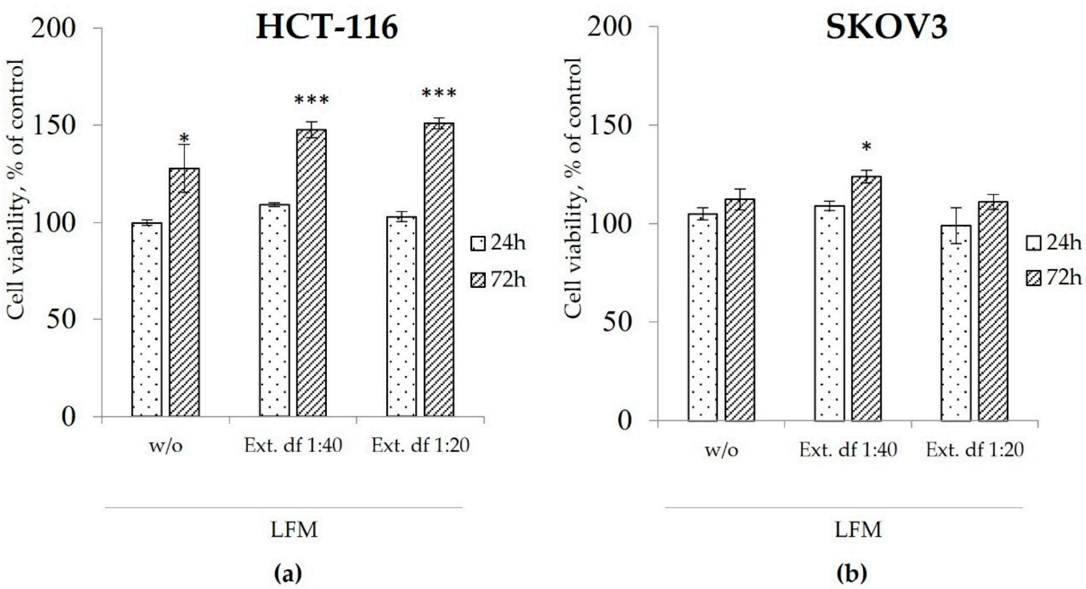

Figure S2. Cell viability of human colorectal carcinoma HCT-116 (a) and ovarian carcinoma SKOV3 (b) cells determined via the MTT assay. Cells were exposed to Lysine-free (LFM) media with or without an Isp-containing extract of *I. spicata* (Ext.) prepared as described in Materials&Methods. The treatment time corresponds to the analytical time points at 24 and 72h as indicated. Two dilutions of the extract were applied that correspond to final concentrations of Isp of ~25μM (df (dilution factor) 1:40) and 50μM (df 1:20), respectively. Data show relative values to the measurements before treatment (time point 0) set to 100% and are presented as mean±SD of three independent experiments. (*P≥0.05,**P≥0.01,***P≥0.001 versus 24h timepoint of each group).
