# Supplementary figures and images for: Extract of Indigofera spicata Exerts Antiproliferative Effects on Human Colorectal and Ovarian Carcinoma Cells
Source: Toxins (Basel). 2025 Aug 29;17(9):431. doi: 10.3390/toxins17090431 (PMC12474036; doi:10.3390/toxins17090431)

Isp

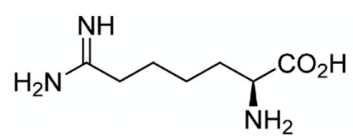

Arg

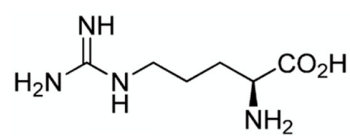

**Figure S1.** Chemical structures of L-indospicine, Isp (left) and L-arginine, Arg (right).

Supplement: Supplementary file 1 [file toxins-17-00431-s001.zip › Figure S1_.pdf]
